# Rapid and non-invasive diagnostic techniques for embryonic developmental potential: a metabolomic analysis based on Raman spectroscopy to identify the pregnancy outcomes of IVF-ET

**DOI:** 10.3389/fcell.2023.1164757

**Published:** 2023-06-23

**Authors:** Hui Meng, Shan Huang, Feiyang Diao, Chao Gao, Jun Zhang, Lingyin Kong, Yan Gao, Chunyan Jiang, Lianju Qin, Ying Chen, Mengna Xu, Li Gao, Bo Liang, Yanqiu Hu

**Affiliations:** ^1^ State Key Laboratory of Reproductive Medicine, Clinical Center of Reproductive Medicine, First Affiliated Hospital, Nanjing Medical University, Nanjing, China; ^2^ Basecare Medical Device Co., Ltd., Suzhou, China; ^3^ State Key Laboratory of Microbial Metabolism, Joint International Research Laboratory of Metabolic and Developmental Sciences, School of Life Sciences and Biotechnology, Shanghai Jiao Tong University, Shanghai, China

**Keywords:** Raman spectroscopy, machine learning algorithm, non-invasive diagnostic techniques, pregnancy outcomes, relative quantitative analysis

## Abstract

The non-invasive and rapid assessment of the developmental potential of embryos is of great clinical importance in assisted reproductive technology (ART). In this retrospective study, we analyzed the metabolomics of 107 samples provided by volunteers and utilized Raman spectroscopy to detect the substance composition in the discarded culture medium of 53 embryos resulting in successful pregnancies and 54 embryos that did not result in pregnancy after implantation. The culture medium from D3 cleavage-stage embryos was collected after transplantation and a total of 535 (107 × 5) original Raman spectra were obtained. By combining several machine learning methods, we predicted the developmental potential of embryos, and the principal component analysis–convolutional neural network (PCA-CNN) model achieved an accuracy rate of 71.5%. Furthermore, the chemometric algorithm was used to analyze seven amino acid metabolites in the culture medium, and the data showed significant differences in tyrosine, tryptophan, and serine between the pregnancy and non-pregnancy groups. The results suggest that Raman spectroscopy, as a non-invasive and rapid molecular fingerprint detection technology, shows potential for clinical application in assisted reproduction.

## 1 Introduction

It is well known that transferring potential embryos is a key part of *in vitro* fertilization (IVF). In the past 30 years, the evaluation of *in vitro* embryos has greatly improved. At present, the main methods used to evaluate embryo potential are morphological evaluation and preimplantation genetic testing (PGT). Morphological evaluation can be divided into traditional static assessment and dynamic time-lapse evaluation. Traditional static assessment is based on static observation related to specific time points in the day. The advantage of this method is that it is simple to perform, but it has obvious drawbacks, such as inter- and intra-observer differences (Paternot, G. et al., 2011). In addition, the embryo implantation rate of this optimized method reaches up to only 40% (Coughlan, C. et al., 2014), which is not satisfactory. The time-lapse monitoring (TLM) technique is a new technique for the evaluation of embryo morphology and developmental dynamics in assisted reproduction. TLM allows continuous image acquisition of early embryonic development so that we can observe the entire developmental process of the embryo. In addition, embryo scoring does not require removing the embryo from the incubator, so it prevents embryo exposure to room temperature conditions. At present, no high-quality data supports that the technology significantly improves clinical outcomes, although TLM may better identify embryos with developmental potential (Kirkegaarad et al., 2015). However, it must be noted that the TLM technique currently involves expensive equipment.

Preimplantation genetic testing for aneuploidy (PGT-A) involves taking a small number of cells from an embryo and using genetic technology to detect those cells with 99.9% accuracy (Capalbo, A., et al., 2015). However, PGT-A is invasive, time-consuming, and complex and can cause potential damage to the embryo if not performed properly. The results of PGT-A do not necessarily equate to genetic information on the entire embryo due to the presence of mosaics. Additionally, trophectoderm biopsy with a relatively high DNA content will reduce the chance of birth (Neal et al., 2017). In addition, biopsy techniques may affect maternal and neonatal outcomes (Popovic, M., et al., 2020) (Zhang et al., 2019) (Alteri et al., 2023). PGT-A increased the live birth rate of blastocyst-stage embryos in female patients who were over 35 years old. However, it did not improve the clinical outcomes of the general population (Simopoulou et al., 2021). Thus, PGT-A is not suitable for all patients, and some patients, namely, those with diminished ovarian reserve, advanced maternal age, or recurrent implantation failure, may have limited access.

Due to delayed childbearing, environmental pollution, and increased psychological stress, an increasing number of patients, including those with diminished ovarian reserve, advanced maternal age, or recurrent implantation failure, are seeking the help of fertility specialists. These patients are clinically characterized as having fewer oocytes and/or lower implantation and clinical pregnancy rates. However, good-quality embryos can also be obtained in patients with poorer prognoses than those with normal ovarian reserve (Wu, W., et al., 2020). The same is true of embryos from patients with repeated implantation failures. The probability of egg chromosome abnormalities increases with increasing female age (Pellestor, F., et al., 2003). Therefore, for these patients with a poor prognosis, the implantation and pregnancy capacity cannot be accurately predicted through conventional embryo assessment methods.

A great deal of research progress has been made in the application of non-invasive detection technology for the evaluation of embryonic cell development. Non-invasive detection techniques commonly used in the developmental metabolomics of embryos include thin-layer chromatography (TLC) (Wiener-Megazi et al., 2011), near-infrared (NIR) spectroscopy (Seli et al., 2011), microfluorescence (Gardner et al., 2011), enzyme-linked immunosorbent assay (ELISA) (Combelles et al., 2012), nuclear magnetic resonance (NMR) spectroscopy (Kirkegaard et al., 2014), and Fourier transform infrared (FTIR) spectroscopy (Muñoz et al., 2014). These techniques can be used to obtain information on metabolic components indirectly and non-invasively, complete a qualitative analysis, and evaluate the developmental quality of embryos. In contrast to the traditional method of subjective evaluation of embryo developmental potential, these technologies can achieve qualitative analysis by quantifying biomarkers. However, these techniques, such as the NIR (Vergouw et al., 2014) and FTIR techniques, also have disadvantages in terms of detecting the solution of the culture medium, the factors influencing the water absorption spectrum, and the need to prepare complicated biochemical reactions in advance. These methods are not only expensive but also inefficient. In clinical applications, there is an urgent need for a non-invasive, fast, and anti-interference technology to complete a qualitative analysis.

The Raman spectrum is a scattering spectrum based on molecular vibration. When the laser irradiates the sample to be measured, the excited light particles exchange energy with the substance molecules, thus causing the frequency change of the light particles. This kind of light is called Raman scattering. The frequency change of the scattered light depends on the energy of the molecular bond, and molecular diagnosis can be made by using this characteristic (Cialla-May et al., 2019). In the field of assisted reproduction, Raman spectroscopy can be used as a metabolic profiling method to assess the development of germ cells. In past studies, Raman spectroscopy was used to identify the chromosome ploidy of embryos (Liang et al., 2019), predict the possibility of developing into blastocysts according to the D3 metabolic medium (Zheng et al., 2021), and research the potential markers of oocyte development in patients with polycystic ovary syndrome according to follicular fluid (Huang et al., 2021; Zhang et al., 2021). In the present study, Raman spectroscopy was used as a non-invasive detection technique to assess the metabolic levels in the culture medium and to determine the physiological state of cells and their potential for further development.

## 2 Materials and methods

### 2.1 Embryo cultivation and development

Female volunteers undergoing IVF treatment at the First Affiliated Hospital of Nanjing Medical University were recruited for this research study. The inclusion criteria were based on the following clinical characteristics: advanced maternal age, diminished ovarian reserve, or repeated implantation failure. All patients underwent mild stimulation or natural cycle treatment. Microdroplet Petri dishes (25 μL/drop) were prepared on the day of oocyte retrieval. The dish was placed in a 37°C, 6% CO_2_ incubator overnight for equilibration. Fertilization was observed 17–18 h after insemination. After 3 days of embryo culture, available embryos were transferred or frozen. Serum *β*-HCG concentrations >25 IU/L at least 14 days following embryo transfer were supposed to be biochemically positive. The presence of an intrauterine gestational sac with positive fetal heart activity at least 6 weeks post-embryo transfer was defined as clinical pregnancy. The continuation of pregnancy over 12 weeks was considered an ongoing pregnancy. Preterm birth was defined as delivery between 28 and 37 weeks of gestation, and term birth was defined as delivery after 37 weeks of gestation; both preterm and term births are referred to as live births.

### 2.2 Clinical information statistics

A total of 107 samples of the discarded culture medium from embryo cell transplantation were collected from participants for this study. The grouping criteria for the statistical data involved classifying samples into two groups: the pregnancy group, which comprised samples with a clinical outcome of live birth after embryo cell transplantation, and the non-pregnancy group, which comprised samples with a negative HCG test result 14 days after embryo transplantation. The 10 clinical characteristics of participants corresponding to the 54 non-pregnancy and 53 pregnancy control samples are summarized in Table 1. One of the features was the statistical analysis of embryo quality, which was classified into two categories—“good” and “poor”—based on morphology. Specifically, cleavage-stage embryos were defined as good quality if they had 6–12 blastomeres on day 3, contained <20% anucleate fragments, had even-sized blastomeres, and exhibited no apparent morphological abnormalities. Cleavage-stage embryos were defined as poor-quality embryos if they were of moderate quality, with less than five cells on day 3 and/or 20–50% fragmentation, or were of poor quality, with less than three cells on day 3 and over 50% fragmentation.

**TABLE 1:** Clinical characteristics of all participants.

		Pregnancy (n = 53)	Non-pregnancy (n = 54)	*p*-value
BMI (kg/m^2^)		22.92 ± 2.87	22.09 ± 2.23	0.114
AMH (ng/mL)		1.36 ± 1.28	1.05 ± 1.00	0.185
AFC		5.04 ± 3.95	3.37 ± 2.31	0.012 *
FSH (IU/L)		11.87 ± 12.43	12.78 ± 8.64	0.691
E_2_ (pmol/L)		241.23 ± 374.42	365.00 ± 446.40	0.159
PRL (ng/mL)		288.17 ± 182.02	329.03 ± 220.21	0.387
LH (IU/L)		6.64 ± 11.35	5.66 ± 4.07	0.588
Patient age (years)		34.45 ± 4.35	35.69 ± 5.61	0.223
Duration of infertility (years)		3.92 ± 2.83	4.40 ± 3.59	0.466
Embryo quality	Good	86.79% (46/53)	87.04% (47/54)	0.598
	Poor	13.21% (7/53)	12.96% (7/54)	0.598

Note: BMI, body mass index; AMH, anti-Müllerian hormone; AFC, antral follicle count; FSH, follicle-stimulating hormone; E2, estradiol; PRL, prolactin; LH, luteotrophic hormone. Data are presented as the mean ± SD. The (*) values indicate the significant differences between the two groups (*p* < 0.05).

### 2.3 Sample collection and preparation

**FIGURE 1:**
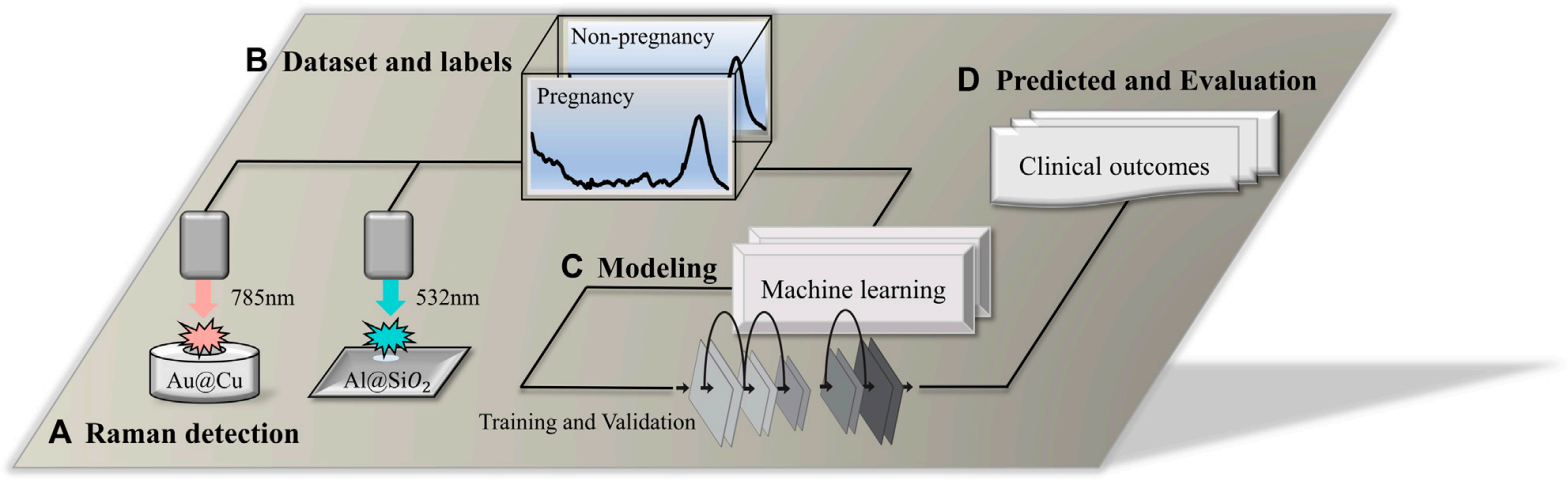
Technical route flow chart. **(A)** Raman detection using a 532-nm laser platform and 785-nm laser platform; the $Al@{SiO}_{2}$ slide and Au@Cu sample pool were used as the sample containers. **(B)** Dataset and labels. Data were labeled based on clinical records. **(C)** Modeling. After data preprocessing, the classification model was established by quantitative and qualitative algorithms. The modeling process includes training and validation. **(D)** Prediction and evaluation. A blind test dataset was developed to test the model's accuracy, and the performance of predicted clinical outcomes was evaluated.

After the removal of the embryos, approximately 20 μL of the culture solution was collected and quickly frozen at −80°C. The samples were transferred to a dry ice storage box (−60∼−57°C) and transported to the Raman laboratory. After removing the sample, it was thawed at room temperature (20–25°C) for 20–30 min. For the crystalline sample, 1.5–2 μL of sample droplets were drawn on the special substrate (Al@ SiO_2_) and placed into a constant-temperature blast drying oven for moisture drying and crystallization (35°C, 25–30 min). For the liquid samples, 7 μL of sample droplets was moved onto the enhanced substrate (Au@Cu), as shown in Figure 1A.

### 2.4 Raman spectroscopy

A confocal Raman microscope (BaseRaman Pro, China) equipped with a 532-nm laser (Nd: YAG, ∼30 mW) was used to detect the crystalline samples, and the laser power focused on the sample was ∼5 mW. An ultrahigh throughput spectrometer (UHTS), CCD camera (DV401-BV), 50× microscope objective (Zeiss EC Epiplan, NA = 0.75), grating of 1200 l/mm, exposure time for single point detection of 4 s, and an accumulation frequency of 15, and 5 single points were randomly selected for spectrum acquisition for each sample. A desktop Raman spectrometer (BaseRaman200, China) was used to detect the original liquid samples. The excitation light source was a 785-nm laser (the maximum laser power was 320 mW), and the sample was incident at 100% laser intensity. As shown in Figure 1B, the data set was acquired using the above two Raman spectrometers. Each sample was detected five times, with three scans performed during each detection using a 40 s exposure time. The average of the three exposure signals was used as the original data. The calibration was based on the characteristic peak of the silicon wafer at 520.5 cm^−1^, and the calibration was within the error range of ±0.15 cm^−1^. The environmental parameters of the instrument were recorded after calibration of the instrument (19.1°C ≤ Ambient temperature ≤23.9°C, 30% ≤Air humidity ≤51%).

### 2.5 Data analysis and model evaluation

Raman spectroscopy is a powerful tool for analyzing the molecular composition of samples. However, raw Raman spectra often contain noise and baseline drift, which can affect the accuracy of subsequent analysis. Therefore, it is necessary to perform data preprocessing on the Raman spectra to improve the signal-to-noise ratio and remove the baseline drift. Several preprocessing methods, such as baseline correction, smoothing, and normalization, have been proposed to enhance the quality of Raman spectra. The data pre-treatment process in this study included eliminating the high-energy cosmic ray interference peak (a sharp characteristic peak with a narrow spectral band and large peak), intercepting the Raman spectral band (region from 600 to 1800 cm^-1^) of the “biological fingerprint,” and using the Savitzky‒Golay filter to smooth the spectrum (to improve the spectral signal-to-noise ratio). Then, combined with statistics-sensitive non-linear iterative peak (SNIP) clipping, the baseline correction of the spectrum was completed by deducting the background of the substrate, fluorescence, and redundant signal contributions. Finally, all the data were normalized to [0, 1].

As shown in Figures 1C, D. For the establishment of a classification model, multiple types of machine learning algorithms were applied to predict the outcome of embryos. The data set was divided according to a ratio of the training set to the test set of 4:1. Before training the model, the principal component analysis (PCA) algorithm was used to extract Raman spectral features, and the data were reduced in dimension. In the present research, 4 supervised learning algorithms, convolutional neural network (CNN), support vector machine (SVM), random forest (RF), and extreme gradient boost (XGBoost), were used to train and establish classification models. For the performance evaluation of the prediction model, methods include calculating the confusion matrix, accuracy, precision, recall, F1-score, receiver operator characteristic (ROC), and area under the curve (AUC) (Pieszko and Slomka, 2021).

### 2.6 Software and programs

The program languages used for data preprocessing, model building, result visualization, and other data analysis were Python 3.6 and R 4.1.0. The tools for program development were PyCharm 2020.1 and RStudio 2022.02.1.

## 3 Results

### 3.1 Data preprocessing

**FIGURE 2:**
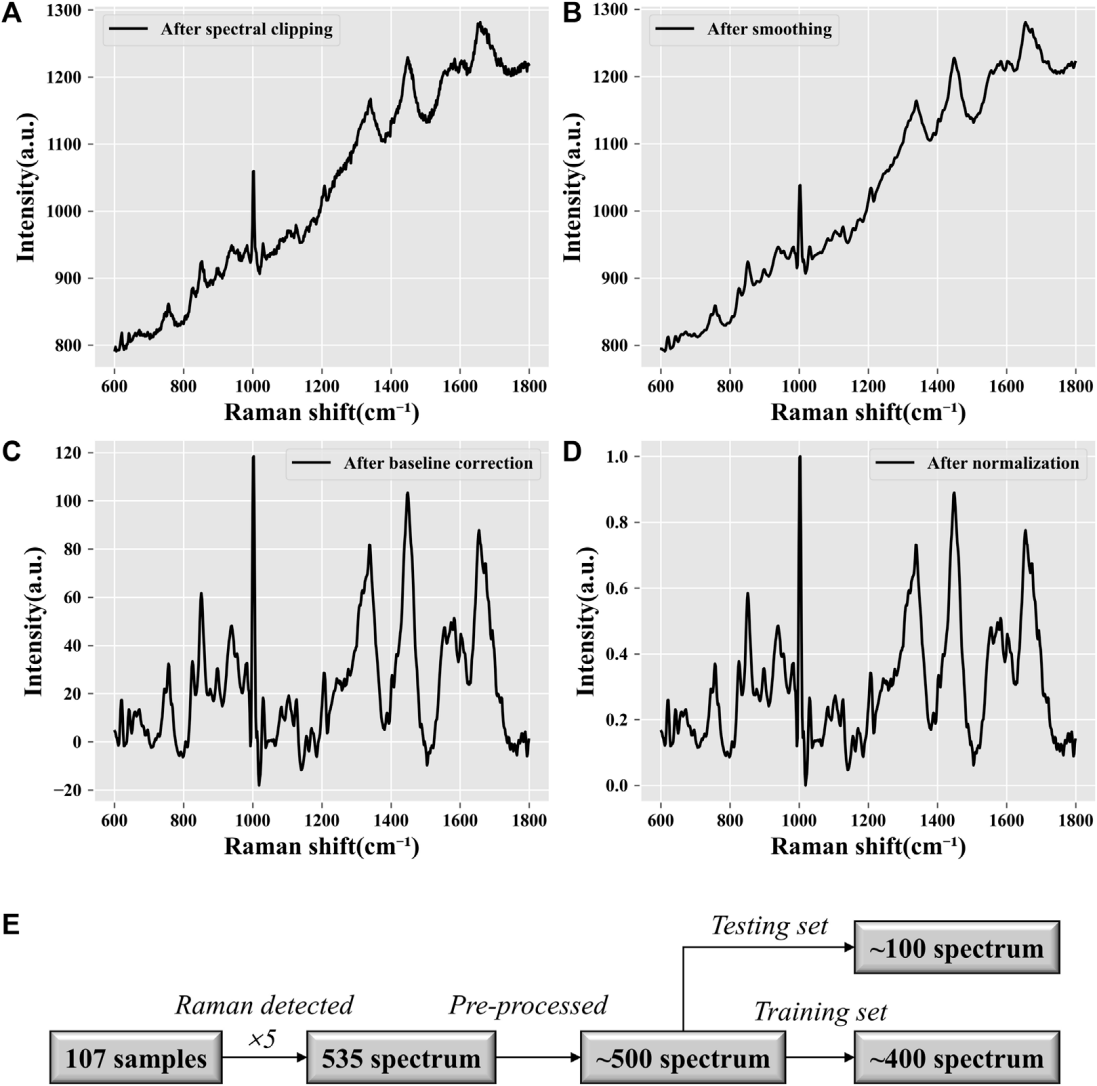
Data preprocessing visualization. **(A)** Spectral clipping. Extraction of the “biological fingerprint region” of Raman spectra. The processed Raman spectra are limited to the range of 600–1,800 cm^−1^. **(B)** Noise reduction. Smoothing filtering can reduce the spectral noise and improve the signal-to-noise ratio. **(C)** Baseline correction. Fitting the background fluorescence signal in the original signal and subtracting it. **(D)** Normalization. Standardizing all data to a range of [0, 1] allows all spectral intensities to be on the same scale. **(E)** Data quantification. Statistical analysis of the data volume.

In this research, the spectral range of the collected raw Raman spectral data was 50–2000 cm^-1^. The visualization results of the data after spectral clipping (Figure 2A), smoothing filtering (Figure 2B), baseline correction (Figure 2C), and normalization (Figure 2D) are shown in Figure 2. After data preprocessing, the initial Raman signals of all samples removed invalid interference information (noise and background fluorescence signals) and were reduced to a unified data scale. As shown in Figures 2A and E, a total of 107 samples were collected and five spectra were repeated for each sample. A total of 535 (107 × 5) Raman spectra were obtained. The abnormal data were eliminated, and the whole dataset was left with approximately 500 spectra from 100 samples. Then, according to the ratio of 4:1, they were divided into a training set and a test set. The training set contained approximately 400 spectrum data points from 80 samples, while the test set contained approximately 100 spectrum data points from 20 samples. The proportion of different pregnancy outcome samples in each dataset was 1:1.

### 3.2 Biochemical analysis and biomarker research

**FIGURE 3:**
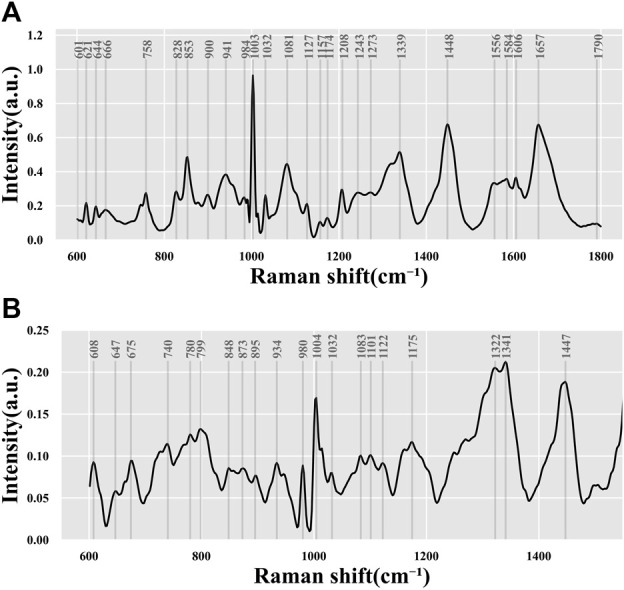
Average spectrum of all data. Data from **(A)** 532-nm Raman platform and **(B)** 785-nm Raman platform.

To obtain the average Raman spectrum, the Raman spectrum data on all collected samples of the embryo culture medium were averaged, as depicted in Figure 3. Analysis of the Raman shift and intensity of characteristic peaks in the Raman spectrum revealed molecular fingerprints that express biochemical information on the culture medium. The characteristic peaks of amino acids, proteins, lipids, nucleic acids, and other molecules in the biological fingerprint region appeared within the Raman frequency shift range of 600–1,800 cm^−1^, as shown in Table 2. By referring to the Raman database used in biological research, we can deduce the molecular vibration modes and material distribution corresponding to different Raman peaks.

**TABLE 2:** Vibration mode and composition distribution of the partial Raman shift position.

Position ( ${cm}^{-1}$ )	Vibration mode and composition distribution	Position ( ${cm}^{-1}$ )	Vibration mode and composition distribution
621	C–C twisting mode of phenylalanine (proteins)	1157	In-plane vibrations of the conjugated = C–C =
644		1174/1175	Tyrosine, phenylalanine, and C–H bend (protein)
666	G, T (DNA bases)-tyrosine-G backbone in RNA	1208	$\nu \left(C-C_{6}H_{5}\right)$ /protein assignment
758	Tryptophan	1243	Amide III
828	O–P–O stretching/phosphodiester	1273	$\delta \left(C=CH\right)$
853	C–C stretch/tyrosine	1339/1341	C–C stretch of the phenyl/nucleic acid mode
900/895	(C–O–C) skeletal mode/glycine/β-glucose	1447/1448	${CH}_{2}{CH}_{3}$ deformation/ ${CH}_{2}$ /C–H vibration
941/934	Skeletal modes/polysaccharides	1556	Amide II
1003/1004	Phenylalanine and C–C skeletal mode	1584	C=C bending mode of phenylalanine
1032	${CH}_{2}{CH}_{3}$ bending modes	1606	C=C bending
1071/1073	Phosphate vibrations (nucleic acids)	1657	C=C/amide I/fatty acids
1127/1122	$\nu \left(C-N\right)$		

The study found that, based on the biochemical information characterized by each characteristic peak in Table 2 and Figure 3, the most intense signal was contributed by the phenylalanine ring symmetrical vibration at 1003/1004 cm^−1^. Other commonly strong signals included C–C bond stretching (853 cm^-1^), carbonate/phosphate or C–C bond vibration (1071/1073 cm^-1^), C–C stretch of the phenyl or nucleic acid (DNA/RNA) mode (1339/1341 cm^−1^) in amino acid, ${CH}_{2}{CH}_{3}$ deformation or ${CH}_{2}$ or C–H vibration (1447/1448 cm^−1^), and C=C or amide I or fatty acids (1657 cm^−1^). Some weak Raman signals, including 621 and 644 cm^-1^ contributed by the C–C twisting mode in phenylalanine, tryptophan (758 cm^−1^), O–P–O stretching/phosphodiester (828 cm^−1^), ${CH}_{2}{CH}_{3}$ bending modes (1032 cm^−1^), and ν(C-N) (1127/1122 cm^−1^), can be found in the Raman database summarized by previous biomedical reports (Talari Sekhar et al., 2015).

The Raman spectrum depicts the vibration distribution of molecules within the complex mixed components. Our study analyzed samples to obtain information on the residual components in the culture medium, following cellular uptake and the products of cellular metabolism. The underlying reasons for variations in cellular metabolism across different cell types are multifaceted. Simple biochemical analyses are not sufficient to identify the physiological state of cells or reflect their developmental potential. Thus, further information mining through advanced algorithms is necessary.

### 3.3 Machine learning classification models

**FIGURE 4:**
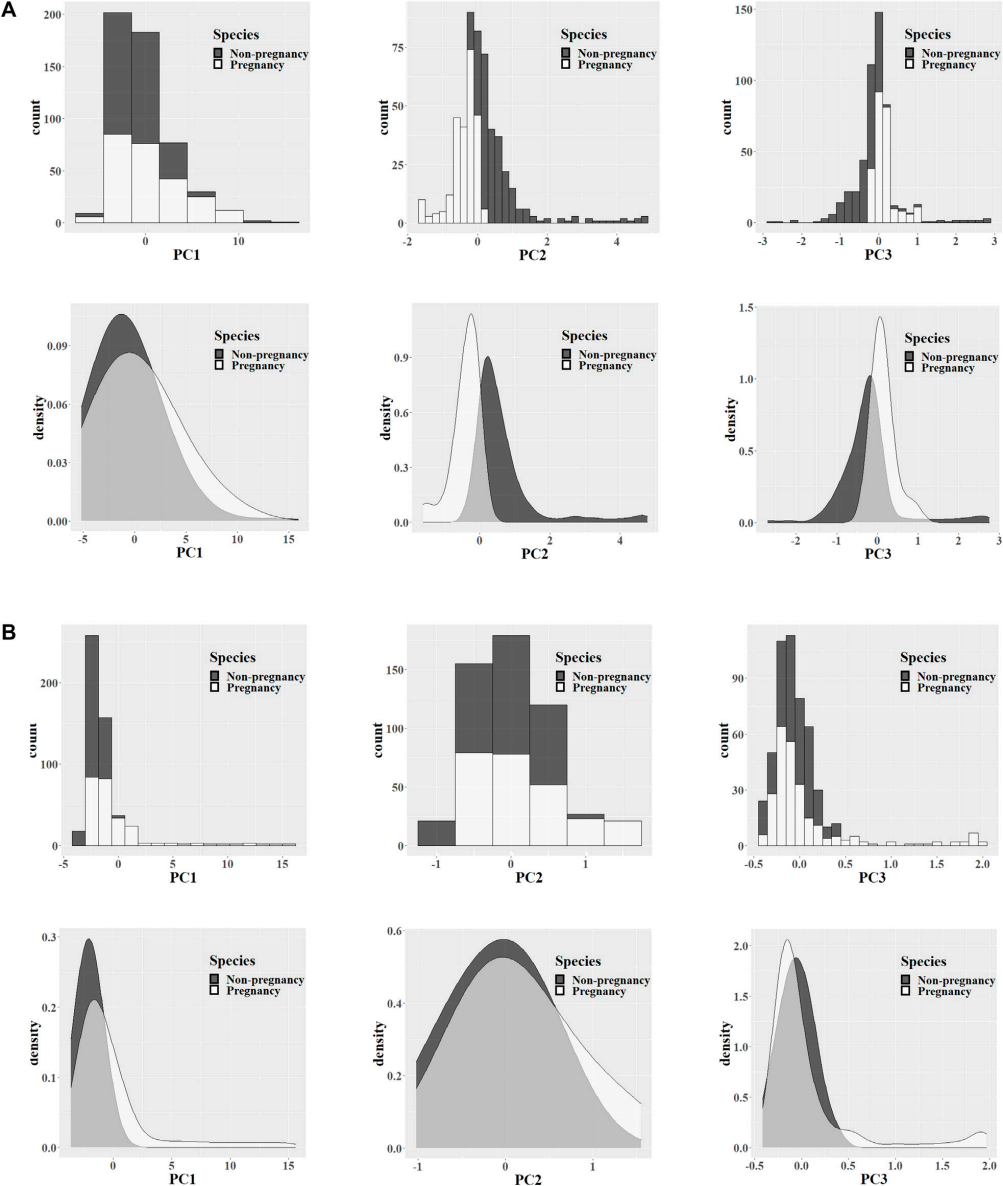
PCA feature analysis. Data from the 532-nm **(A)** and 785-nm **(B)** experiment platforms. Extracting the Raman feature principal components of the pregnancy and non-pregnancy groups, counting the first three principal component values, and exhibiting the histogram and nuclear density map of the feature distribution.

After processing the original data, there were still approximately 600–700 discrete feature values in the biometric fingerprint region of the Raman spectrum. Such high-dimensional Raman spectrum data usually contain a large amount of redundant information, which is not conducive to finding the differences between different species, so it was necessary to reduce the dimension of the data. Calculated by the PCA algorithm, the first three principal components of each spectrum after data dimensionality reduction were extracted, the counts of different principal components were counted, and a histogram and a kernel density map were drawn. As shown in Figure 4A, the crystalline samples detected by the 532-nm instrument platform had more obvious differences in Raman characteristics between the different groups. It may be that crystalline samples with higher concentrations of substances can express more metabolite differences, and the second and third principal components have more significant differences. (B) The liquid culture medium sample detected by the 785-nm instrument platform had a low material concentration, so the differences in expressed metabolites were not significant enough. The first three principal components showed only small differences in characteristics.

Unsupervised analysis can extract hidden information from large-scale datasets, and it is not difficult to find the distribution differences of their features through clustering. However, this did not directly distinguish the attributes of the sample, so it was necessary to further combine supervised learning algorithms to establish a classification model. Before modeling, we reduced the dimensions of the data involved in the modeling and extracted the first 100 principal components. It not only avoided the overfitting of redundant data but also sped up the training. In the external blind test datasets, 95 and 94 spectra participated in the evaluation of the pregnancy outcome model of the 532-nm and 785-nm platforms, respectively. Compared with other machine learning methods, the one-dimensional convolutional neural network (1D-CNN) model can achieve higher prediction accuracy, with the accuracy of predicting pregnancy outcomes reaching 71.5% and 67.85%, respectively. In addition, we evaluated the performance of the model by several measure indexes, such as precision, recall, and F1-score, which were calculated according to the confusion matrix. The prediction results of pregnancy outcome and performance evaluation of the PCA-CNN model are shown in Table 3.

**TABLE 3:** Prediction results and performance evaluation of the PCA-CNN model. Model of the (A) 532-nm platform and (B) 785-nm platform.

A	Confusion matrix		Performance evaluation			
Species	Predicted		Precision	Recall	F1-score	Accuracy
	Pregnancy	Non-pregnancy				
Pregnancy	*31*	*14*	*0.70*	*0.69*	*0.70*	*68.89%*
Non-pregnancy	*13*	*37*	*0.73*	*0.74*	*0.73*	*74%*
Total					*71.5%* (*68/95*)	

B	Confusion matrix		Performance evaluation			
Species	Predicted		Precision	Recall	F1-score	Accuracy
	Pregnancy	Non-pregnancy				
Pregnancy	*28*	*15*	*0.65*	*0.65*	*0.65*	*65.12%*
Non-pregnancy	*15*	*36*	*0.71*	*0.71*	*0.71*	*70.59%*
Total					*67.85%* (*64/94*)	

### 3.4 Relative quantitative analysis of amino acids

In recent years, there has been increasing interest in studying the correlation between the quality of IVF embryo development and the metabolites in the culture medium. Several studies have been conducted to investigate the relationship between the metabolic activity of embryo cells and their developmental potential. The study by Seli et al. (2008) used proton nuclear magnetic resonance (NMR) spectroscopy to analyze the metabolites in the culture medium of human embryos. The study found that certain metabolites, such as glucose and pyruvate, were associated with embryo quality and could be used as predictors of pregnancy outcomes. The study highlighted the potential of NMR spectroscopy as a non-invasive tool for assessing embryo quality. In the study by Inoue et al. (2021), metabolomic analysis was performed on the culture medium obtained from a single blastocyst that was cryopreserved after culturing. A total of 469 metabolites were identified, of which 187 (39.8%) were organic acid metabolites. Significant changes (*p* < 0.05) were observed in eight metabolites between the high-quality embryo group and the low-quality embryo group. Differences in several metabolic pathways were found between the high-quality and low-quality embryo groups, with the significantly changed metabolites involving mainly the metabolism of branched-chain amino acids. The study by Xia L. et al. (2014) used gas chromatography/mass spectrometry (GC/MS) to analyze the metabolites in the follicular fluid and oocyte culture medium of women undergoing IVF treatment. The study found that certain metabolites, such as amino acids and lipids, were associated with oocyte developmental competence and could be used as biomarkers for predicting IVF outcomes. The study highlighted the potential of GC/MS as a tool for identifying biomarkers of IVF success and developing personalized treatment strategies for women with infertility. Further research in this area could lead to the development of new diagnostic tools and personalized treatment strategies for infertility.

**FIGURE 5:**
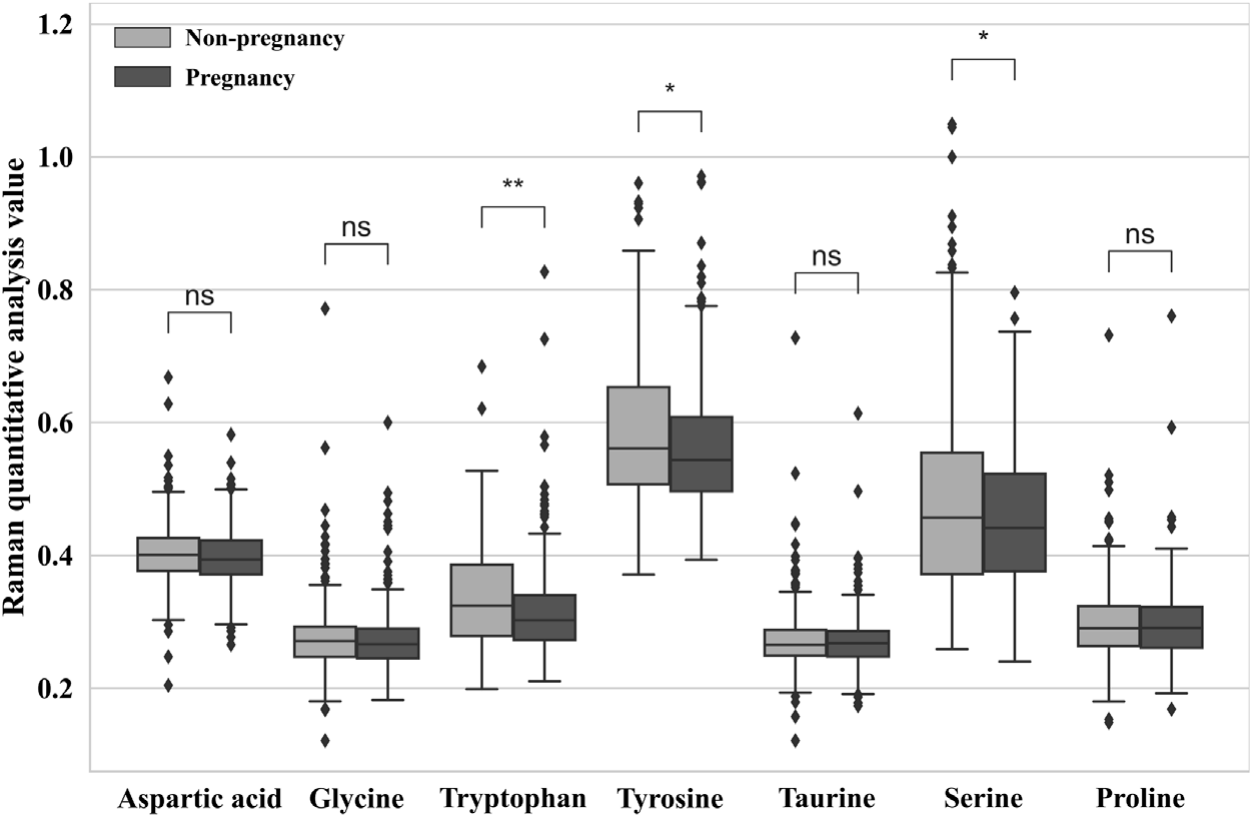
Relative quantitative ratio value of different embryo outcomes. (*p < 0.05 = significant difference, **p < 0.01 = extremely significant difference, and ns = not significant.)

This research paper used Raman spectroscopy and internal standard quantification methods to present a statistical analysis of the metabolic levels of seven amino acid components in discarded culture media of embryonic cells with and without pregnancy potential. The internal standard quantification algorithm used the characteristic peak intensity of a biomarker as the expression value $I_{targets}$ for molecular concentration and the characteristic peak of a standard substance in the detection system as the expression value $I_{standards}$ for internal standard molecular concentration. The quantitative ratio was obtained from Equation 1. The seven amino acids analyzed were aspartic acid, glycine, tryptophan, tyrosine, taurine, serine, and proline. These were quantified based on the peak intensity of the biomarker characteristic peaks at 941 cm^-1^, 900 cm^-1^, 758 cm^-1^, 853 cm^-1^, 1032 cm^-1^, 1326 cm^-1^, 828 cm^-1^, and the internal standard characteristic peak at 1003 cm^-1^. For the complex mixing system, the characteristic peak with the most intense molecular vibration of each amino acid was chosen as the quantitative analytic value. For example, the characteristic peaks of aspartic acid, taurine, and serine at 941, 1032, and 1326 cm^-1^ belonged to the C–H bending vibration, S=O stretching vibration, and C–N stretching vibration, respectively (Wang et al., 2022). The characteristic peak at 900 cm^-1^ of glycine could be assigned to the scissoring and stretching vibration of $\delta \left(COOH\right)$ and $\nu \left(C–C\right)$ (Tanaka, et al., 2017). The peaks observed at 758, 853, and 828 cm^-1^ for tryptophan, tyrosine, and proline, respectively, could be attributed to the bending vibrations of aromatic C–H and C–C–C bonds (Socrates, 2004). The results of the quantitative analysis with the specificity of the molecular vibration mode are shown in Figure 5. All spectral data from pregnancy and non-pregnancy groups represent the levels of metabolism of the seven amino acids by embryos capable of developing to pre-densification. The results showed that there were differences in three amino acids (i.e., tyrosine, tryptophan, and serine), while no significant differences were noticed in the other four amino acids. The differences in amino acid components obtained by Raman characteristic analysis expressed the amino acid metabolism levels of embryos with different pregnancy outcomes. It can be proven that some amino acid metabolism levels may be used to represent different physiological states.

$$Quantitative\;ratio\;value=\log\left(I_{targets}/I_{standards}\right).$$ (1)

### 3.5 Performance evaluation of different classification algorithms

There are differences in the performance of different classification algorithms. Machine learning models are widely used in various fields, including image recognition, natural language processing, and speech recognition. However, the performance of different models varies, and it is necessary to evaluate the performance of different models to select the most suitable model for a specific task. Performance evaluation can help researchers understand the strengths and weaknesses of different models and improve their performance. There are several commonly used performance evaluation metrics, including accuracy, precision, recall, F1-score, and the area under the curve (AUC). These metrics can be used to compare the performance of different models and select the best model for a specific task. For example, if the goal is to minimize false positives, precision may be the most important metric to consider. In addition to selecting the best model, performance evaluation can also help researchers optimize model parameters and improve model performance. By evaluating the performance of different models under different parameter settings, researchers can identify the best parameter values for a specific task (GéRon., 2017).

**FIGURE 6:**
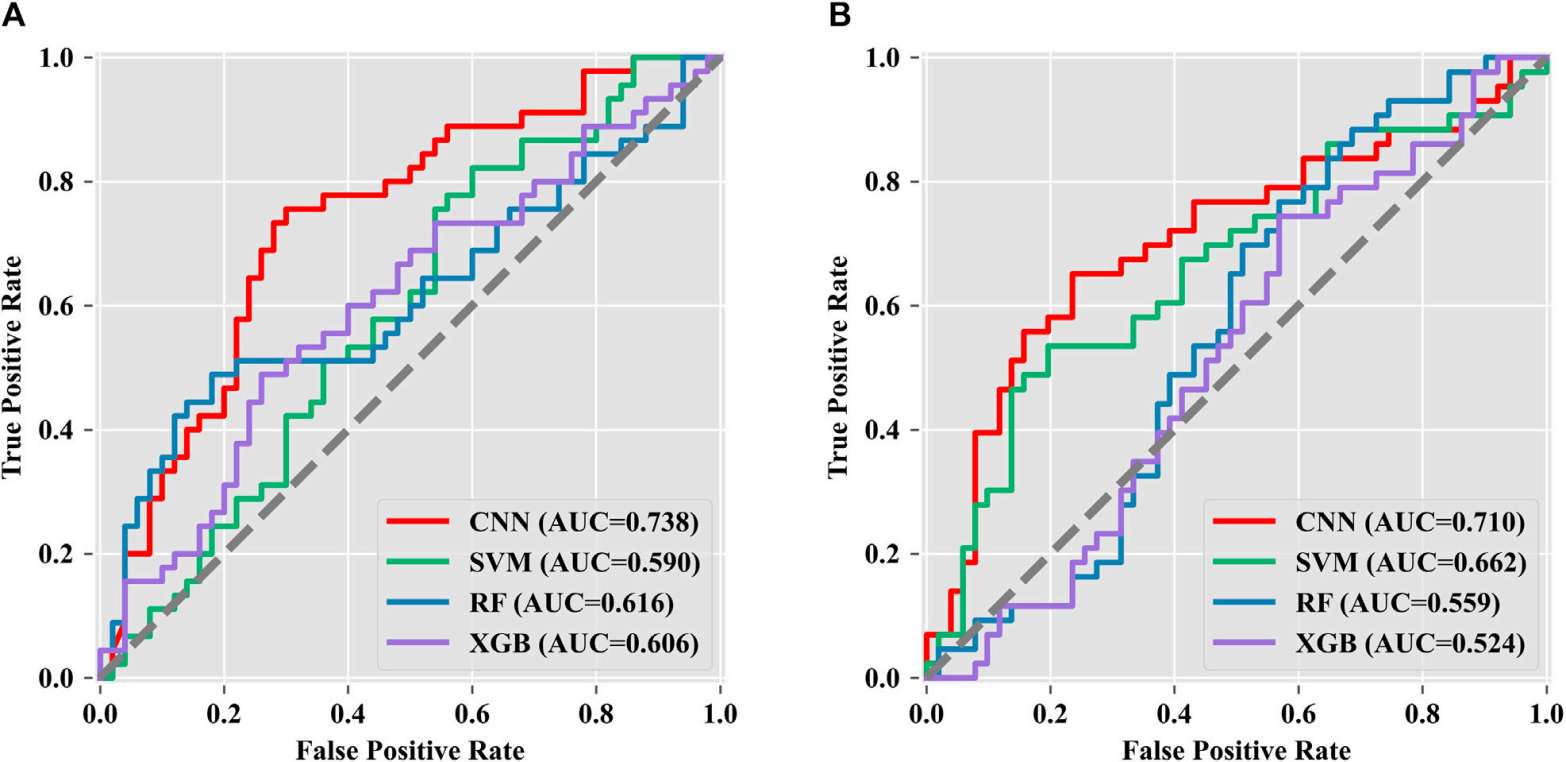
Multi-classifier ROC and AUC evaluation. Data from the **(A)** 532-nm platform and **(B)** 785-nm platform.

In the present study, PCA was used to reduce data dimensionality, and the resulting datasets were used to build artificial intelligence classification models for predicting pregnancy outcomes on the 532-nm and 785-nm platforms using CNN, SVM, RF, and XGBoost algorithms. The predicted accuracies are shown in Table 4, revealing a significant difference in the blind test accuracies of the different algorithms. Among the models developed for the 532-nm platform, the CNN algorithm achieved an accuracy of up to 71.5%, while for the 785-nm platform, the CNN model had an accuracy of 67.85% in predicting pregnancy outcomes. These results demonstrate that the CNN algorithm outperforms the other algorithms. By comparing the modeling results of the data generated from the two platforms, we found that data from the 532-nm platform can be used to establish a model with better classification performance. The performance of the four classifiers was assessed using ROC and AUC, as depicted in Figures 6A, B, for the 532-nm and 785-nm platforms, respectively. The results suggest that for the 532-nm platform, the CNN classifier outperformed the other classifiers. On the other hand, for the 785-nm platform, the CNN classifier performed the best, followed by the SVM classifier, while the other two classifiers (RF and XGB) exhibited poor performance.

**TABLE 4:** Performance of models with different algorithms.

Algorithm	Optimum accuracy of various models	
	532-nm platform	785-nm platform
CNN	(68/95) 71.50%	(64/94) 67.85%
SVM	(53/95) 55.79%	(59/94) 62.77%
RF	(62/95) 65.26%	(53/94) 56.38%
XGBoost	(58/95) 61.05%	(48/94) 51.06%

## 4 Discussion

In clinical applications, pre-embryo transfer screening is generally used to select the D3 cleavage-stage embryo or D5–6-stage blastocyst using a morphological grading evaluation system (Martínez, M. et al., 2018) or morphological kinetics. Embryo cells in different stages have different clinical characteristics. Furthermore, as is the case in patients with normal ovarian function, transplantable embryos and high-quality embryos can also be obtained in patients with poor prognoses (Wu, W., et al., 2020) (Boucret, L., et al., 2022). For those patients, selecting D3 cleavage-stage embryos for transplantation can avoid the risk of embryo-free transplantation and has a lower cost (Maxwell, S. M. et al., 2015). Furthermore, the transfer of blastocyst-stage embryos was associated with shorter LTL in children than was the transfer of cleavage-stage embryos (Wang, C., et al., 2022). However, the accuracy of the assessment of the embryonic development potential of the cleavage-stage before transplantation is low. Aneuploidy is highly associated with embryo failure. In addition, at least 35%–40% of euploid embryos still fail to implant due to metabolic or other non-genetic viability parameters (Scott, RT., et al., 2013). Embryo development and metabolism based on Raman spectra can explore biomarkers that affect embryo development by analyzing the difference in vibration modes of substance molecules (Lima, C. et al., 2021). Previous reports have confirmed that the nutrient substrates and metabolites consumed by embryonic cells in the developmental process are strongly related to embryo outcomes. These studies indicated that the physiological state of cell development could be reflected by metabolic levels (Gardner and Wale, 2013).

Understanding and providing these nutrients in the culture medium is vital for supporting the early development of human embryos in patients treated by assisted reproductive technology. Nutrients such as amino acids (e.g., Gly, Arg, and Leu) and fatty acids have been shown to play a crucial role in oocyte maturation, which involves an increased metabolic rate and oxidative metabolism. The utilization of amino acids also reflects the developmental competence of oocytes in early embryonic development (Gao, H., 2020). Van Winkle reviewed a large body of literature on the impact and related mechanisms of amino acid transporters/transport systems and metabolism on early embryonic development (Van Winkle, 2021). There are still many aspects that need to be clarified regarding the mechanisms by which amino acids affect embryonic cell development. However, current research seems to indicate that certain amino acids play a crucial role in determining the quality of embryonic development.

In this study, we observed significantly higher levels of tryptophan, tyrosine, and serine in the spent culture media of the non-pregnancy group than in the pregnancy group. Our findings are consistent with a previous study by Olcay et al. (2022), which reported that the concentration of tyrosine was significantly higher in aneuploid embryos than in euploid embryos. The authors speculated that chromosomal abnormalities may lead to altered transcription related to protein metabolism, resulting in metabolic alterations in the blastocyst. Notably, Olcay et al. analyzed blastocyst culture media, which is different from our study, suggesting that changes in tyrosine concentration in culture media may occur before blastocyst formation. Our results are also consistent with previous reports that the content of tryptophan and serine differs between embryos that develop from the 8-cell stage to blastocyst and those that arrest before blastocyst formation (Houghton et al., 2002). However, further research is needed to fully understand the possible mechanisms underlying the relationship between amino acid profiles and embryonic development potential.

Raman spectroscopy data analysis combined with machine learning algorithms has been widely used in various qualitative analysis scenarios (Dustin W et al., 2017). In terms of model optimization, it is mainly to adjust the calculation rules by modifying the hyperparameters in the algorithm to adapt to the data characteristics in different scenarios and maximize the accuracy to complete the classification task (Yang and Shami, 2020). In our study, the CNN model needs to consider the number of convolution layers and the number of convolution kernels in each convolution layer because these two parameters have a strong influence on the performance of the model, followed by the number of neurons in the fully connected layer and the selection of the loss function. In addition, the hyperparameters that have a greater impact on the performance of SVM include the selection of kernel functions, penalty coefficients, and parameters of kernel functions. Because it is a classification tree model, the classification performance of the RF algorithm depends on the number of trees, the maximum depth of the tree, the minimum number of samples split, the minimum number of samples on the split leaf nodes, and the randomly selected number of features to build the tree structure. The XGBoost algorithm is also a classifier with a tree structure, but its parameters are different from those of a random forest, such as the maximum number of iterations, the iteration step, the maximum depth of the tree, the proportion of random sampling, and the descending value of the minimum loss function. To find the best parameters, many computing resources are needed for searching. Our experimental results show that model optimization can effectively improve the performance of the classifier. By modifying the parameter values input in the algorithm, the established model can obtain different classification effects. We used the grid search–cross-validation method to find the best parameters and took the final prediction accuracy rate as the standard for evaluation. The 1D-CNN model could extract feature information more effectively through convolution calculation, and it may be more suitable to solve the classification problem of high-dimensional complex Raman spectrum data by recording the data features of different labels with multilayer perceptual neuron weights.

Although our research could reveal some trends, there are still shortcomings. For example, due to the limited number of clinical samples, the generalization performance of the present model has yet to be improved. The remedial work can enhance the robustness of the model by continuing to collect clinical samples and expanding the Raman database. On one hand, the application of algorithms plays an important role in the process of data analysis, and the optimization of algorithms could also compensate for the shortcomings in the current work. On the other hand, the discovery of more significant biomarkers and their quantitative analysis by Raman characteristics may prove more convincing in future clinical applications.

## 5 Conclusion

In this research, a total of 107 samples of the embryo medium were collected and analyzed by Raman spectroscopy to evaluate the metabolic levels of embryo development. The proposed quantitative analysis method based on Raman spectroscopy was applied to assess the metabolic levels of seven amino acids in the D3 culture medium of *in vitro* fertilization embryos. The results indicated significant differences in the metabolism of tyrosine, tryptophan, and serine between pregnancy and non-pregnancy embryo culture media, while no significant changes were observed in the other amino acids. In addition, we adopted four machine learning algorithms, and based on the data obtained from the 532-nm and 785-nm experimental platforms, we established a prediction model of embryo pregnancy results. The prediction accuracy of the PCA-CNN model for the 532-nm platform reached 71.5%, and for the 785-nm platform, it reached 67.85%.

## Data Availability

The raw data supporting the conclusion of this article will be made available by the authors, without undue reservation.
